# Antagonistic Actions of HLH/bHLH Proteins Are Involved in Grain Length and Weight in Rice

**DOI:** 10.1371/journal.pone.0031325

**Published:** 2012-02-21

**Authors:** Dany Heang, Hidenori Sassa

**Affiliations:** Graduate School of Horticulture, Chiba University, Chiba, Japan; National Taiwan University, Taiwan

## Abstract

Grain size is a major yield component in rice, and partly controlled by the sizes of the lemma and palea. Molecular mechanisms controlling the sizes of these organs largely remain unknown. In this study, we show that an antagonistic pair of basic helix-loop-helix (bHLH) proteins is involved in determining rice grain length by controlling cell length in the lemma/palea. Overexpression of an atypical bHLH, named *POSITIVE REGULATOR OF GRAIN LENGTH 1* (*PGL1*), in lemma/palea increased grain length and weight in transgenic rice. PGL1 is an atypical non-DNA-binding bHLH and assumed to function as an inhibitor of a typical DNA-binding bHLH through heterodimerization. We identified the interaction partner of PGL1 and named it ANTAGONIST OF PGL1 (APG). PGL1 and APG interacted *in vivo* and localized in the nucleus. As expected, silencing of *APG* produced the same phenotype as overexpression of *PGL1*, suggesting antagonistic roles for the two genes. Transcription of two known grain-length-related genes, *GS3* and *SRS3*, was largely unaffected in the *PGL1*-overexpressing and *APG*-silenced plants. Observation of the inner epidermal cells of lemma revealed that are caused by increased cell length. *PGL1*-*APG* represents a new grain length and weight-controlling pathway in which *APG* is a negative regulator whose function is inhibited by *PGL1*.

## Introduction

Grain size is an important yield component, and thought to be partly controlled by sizes of glumes; the lemma and palea [1]–[3]. Enlargement of these organs results in a bigger grain, when grain-filling ability remains unchanged. To date, two genes controlling lemma/palea length and two genes involved in grain width have been isolated and characterized. A major QTL (quantitative trait locus) for grain length, *GS3*, encodes a protein with four different domains including the plant-specific organ size (OSR) domain. This domain is necessary and sufficient to function as a negative regulator of rice grain length [4]. Another grain length gene *Small and round seed 3* (*SRS3*) encodes a kinesin13 protein. Constitutive expression of *SRS3* rescued and complemented the short grain phenotype of an *srs3* mutant, suggesting a positive role for *SRS3* in rice grain length [5]. A QTL for seed width on chromosome 5, *qSW5*, was shown to control cell numbers of the lemma and palea, and identified to be functional nucleotide polymorphisms (FNP) for a putative nuclear protein [1]. A QTL for grain width on chromosome 2, *GW2*, encodes a RING-type ubiquitin E3 ligase and controls cell numbers of the lemma and palea [2]. Although grain size is controlled by a complex genetic network in which the above mentioned four genes are also involved, it is expected that many genes are yet to be identified.

Basic helix-loop-helix (bHLH) proteins are the second largest class of plant transcription factors [6]. They comprise two distinct functional regions, a basic region and a helix-loop-helix. The former is required for DNA binding whereas the latter is needed for protein dimerization [7], [8]. Based on DNA-binding ability, the proteins are divided into two groups, 1) DNA-binding bHLH and 2) non-DNA-binding bHLH (HLH) also known as atypical bHLH [9]. It is likely that atypical bHLH function to inhibit bHLH from binding to DNA through heterodimerization [8], [10]. A typical bHLH protein, PIF3, encoded by *Phytochrome Interaction Factors 3*, can bind to the G-box motif (CACGTG) in the promoter region of target genes and is involved in light signaling [11]. In contrast, an atypical bHLH protein, HFR1 (long hypocotyl in far-red), is unable to bind either phytochrome A or B proteins. However, HFR1 modulates phytochrome signaling through heterodimerization with PIF3 [12].

Recent studies have revealed antagonistic roles of HLH/bHLH proteins in various plant organ sizes. For instance, an antagonistic pair of bHLH proteins, *Increased Leaf Inclination* (*Ili*) and *ILI1 binding bHLH* (*OsIBH1*), controls cell length in the lamina joint and leaf bending in rice. Likewise, an *Ili* homolog of Arabidopsis, *Paclobutrazol Resistance1* (*PRE1*), and *AtIBH1* regulate cell elongation [13]. *Activation-tagged bri1 suppressor 1-Dominant* (*atbs1*-D) and *ATBS1 interaction factor*s (*AIF*s) regulate leaf cell size through a brassinosteroid signaling pathway [14].

It is predicted that there are 167 bHLH genes in Arabidopsis, 177 in rice, 99 in poplar, 190 in moss and 13 from five algae species [9], [15]. Despite its vital role, the function of rice bHLH is poorly understood; so far, only ∼10% (19 of 177 genes) of genes have been characterized in rice, compared to 38% (64 genes) in Arabidopsis. In this study, we overexpressed an atypical bHLH gene named *POSITIVE REGULATOR OF GRAIN LENGTH 1* (*PGL1*) in rice lemma/palea and found increases in the length and weight of the grain. We identified a typical bHLH protein named ANTAGONIST OF PGL1 (APG) as an interaction partner of PGL1 and the complex of them is localized in the nucleus. Silencing of *APG* by RNAi resulted in the same grain phenotype overexpression of *PGL1*. Our results suggest that *PGL1* and *APG* antagonistically regulate rice grain length and weight by controlling cell elongation in lemma/palea through heterodimerization.

## Results

### Overexpression of an atypical *PGL1* increases grain length and weight

**Figure 1:**
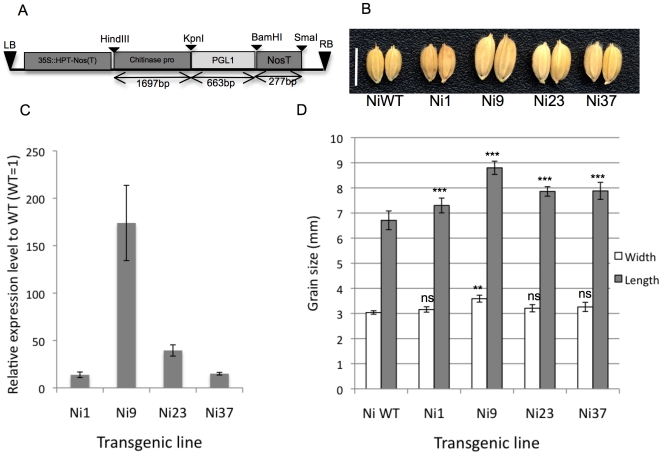
Overexpression of *PGL1* increased grain size in rice. A) Structure of the chitinase promoter and *PGL1* gene in the pPZP2H-lac binary vector. B) Grain phenotype of T_0_ transgenic plants (Ni#) compared with the Nipponbare wild type (NiWT) (bar = 1 cm) C) Quantitative PCR expression analysis of *PGL1* in the lemma/palea of T_0_ plants compared with the wild type (WT = 1) normalized by *OsActin*. Error bar indicates ±sd over three biological repeats. D) Comparison of grain length and width of transgenic T_0_ and wild type plants (error bar, ±sd, n = 10). Asterisks denote a significant difference from the wild type as determined by Student's t tests (ns, not significant; **, p<0.01; ***,p<0.001).

Os03g0171300 (LOC_Os03g07510 in MSU Rice Genome Annotation Project) is an atypical bHLH gene that is not included in the predicted 177 bHLH genes of rice [9], [15], and is reported to be homologous to tomato *Style2.1*, a gene that controls style cell length in tomato [16], at a level of 66% and 63% amino acid identity for the whole sequence and HLH domain, respectively (Figure S1). Analyses of a rice homolog of Os03g0171300 and *Style2.1*, *Ili1*, showed that it is involved in cell length in determining lamina joints of rice [13]. Based on sequence homology, they detected six homologs of *Ili1* in the rice genome, and called one of them, Os03g0171300, *Ili6* [13]. Most members of the ILI family belong to subfamily 16 of the atypical bHLH protein family [15], however, the function of Os03g0171300/ILI6 has not been elucidated yet. We named Os03g0171300 as *POSITIVE REGULATOR OF GRAIN LENGTH 1* (*PGL1*, see below), and analyzed its expression by RT-PCR. The result showed that it is expressed in the pistil, lemma/palea, young panicle, and predominantly in root but not leaf (Figure S1). To analyze the function of *PGL1*, we overexpressed the gene by using a rice chitinase promoter, which was reported to induce gene expression predominantly in rice florets especially in pistils [17] (Figure 1a). Twenty-four and nine independent T_0_ transgenic lines overexpressing *PGL1*, PGL1:OX lines, were produced for two cultivars, Nipponbare and Kita-ake. Although the expression of *PGL1* was significantly increased in pistils, it didn't affect pistil length (Figure S1).

We observed that grain sizes of T_0_ PGL1:OX lines are larger than those of wild types. We examined if the expression level of *PGL1*in lemma/palea correlates with the size of grains. Four representative T_0_ PGL1:OX lines from both backgrounds were selected for analysis. As expected, expression of *PGL1* in lemma/palea was increased and correlated to grain size in the T_0_ transgenics (Figure 1b–d). Quantitative PCR (qPCR) analysis revealed that line Ni9 (T_0_) accumulating 170-fold more of the *PGL1* transcript in lemma/palea showed a 43% increase in 1000-grain weight, while line Ni1 with a 13-fold increase in *PGL1* showed 3% increase in grain weight (Table 1). The large grain size is most probably caused by increased grain length rather than width (Figure 1d). The same results were obtained in Kita-ake background transgenics (Figure S2). The transgenic Ni9 (T_0_) plant with the largest grain was self-pollinated and ten segregated plants were randomly selected for further analysis. The Ni9 (T_1_) plants which were T-DNA-positive remained long whereas the T-DNA negative plant showed a grain length comparable to the wild type (Figure S4). Taken together, it was shown that overexpression of the *PGL1* gene in lemma/palea increases grain length and weight in rice.

**Table 1:** Grain traits and lemma inner epidermis cell of PGL1:OX and APG RNAi lines.

Line	1000-grain weight	Grain length^a^	Grain width^b^	Cell length^c^	Cell width^d^
	(g)	(mm)	(mm)	(µm)	(µm)
NiWT	23.2(100%)	6.9±0.3	2.8±0.2	102.2±20.6	43.7±6.8
Ri-1	26.4(114%)	7.9±0.1***	3.0±0.1*	140.6±23.7***	42.7±5.4*
Ri-2	25.3(109%)	7.7±0.1***	3.0±0.1^ns^	-	-
Ri-9	24.5(106%)	7.8±0.1***	3.0±0.1^ns^	-	-
Ri-12	26.0(112%)	8.0±0.2***	3.1±0.1*	145.9±28.1***	43.8±6.0^ns^
Ni1	23.9(103%)	7.3±0.3***	3.2±0.1^ns^	-	-
Ni9	33.4(144%)	8.8±0.3***	3.6±0.1*	155.1±28.4***	47.2±6.9***
Ni23	26.1(113%)	7.9±0.2***	3.2±0.1^ns^	121.7±26.5***	45.2±5.7**
Ni37	26.0(112%)	7.9±0.3***	3.3±0.2^ns^	128.6±29.4***	44.6±5.3*

a,b: data are the average of 10 samples (±sd).

c,d: data are the average of 250 samples (±sd).

ns , none-significant;

*p<0.05;

**p<0.01;

***p<0.001.

NiWT, Nipponbare wild type.

Ri#, APG RNAi line.

Ni#, PGL:Ox line.

### Interaction between PGL1 and a typical bHLH protein, APG

PGL1 is an atypical bHLH and lacks the basic domain required for DNA-binding, suggesting that it would heterodimerize with other DNA-binding bHLH proteins and abolish their functions as in the case of human Inhibitor of DNA binding (Id) proteins [10]. In order to search for interaction partners of PGL1, we adopted information from the protein-protein interaction network of Arabidopsis. First, we identified an Arabidopsis protein with an HLH motif very similar to that of PGL1. A BLAST search for Arabidopsis proteins (http://www.arabidopsis.org) using the HLH region of PGL1 as a query revealed that PGL1 is highly homologous to Arabidopsis *KIDARI* (KDR, At1g26945) which has 77% and 75% identity with PGL1 for the whole amino acid sequence and HLH domain, respectively. *KDR* was reported to interact with *HFR1* (*bHLH026*, At1g02340) [18]. Then we used the bHLH domain of HFR1 to search the rice genome (http://rapdb.dna.affrc.go.jp/) and found several bHLH proteins as candidates for interaction partners of PGL1. Proteins with *E*-values of <4e^−12^ were selected for analysis; Os12g0610200, Os01g0286100, Os05g0139100, and Os04g0618600. Except for Os04g0618600, all candidates contained amino acids conserved in the basic domain required for binding to DNA [9]. We found expression in the lemma/palea of these candidates. Thus, we chose these four candidates for analysis of interaction with PGL1. For Os05g0139100, the size of the cDNA isolated from lemma/palea (1299 bp for the coding sequence) was different from that of the reported sequence (1518 bp for the coding sequence and 1791 bp for the full-length cDNA, AK287958), probably because of alternative splicing, although we found no longer band corresponding to AK287958 in all the organs analyzed by RT-PCR experiment (Fig. S6). We named Os05g0139100 as *ANTAGONIST OF PGL1* (*APG*, see below), and deposited the cDNA sequence derived from lemma/palea in DDBJ/Genbank/EMBL under accession number AB667900.

**Figure 2:**
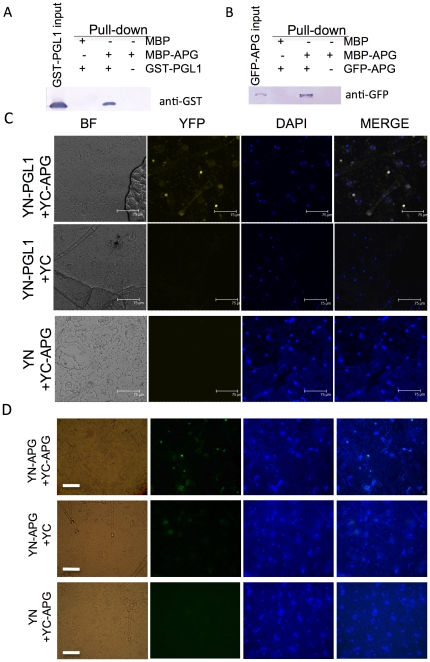
Interaction between PGL1 and APG. A) Interaction between PGL1 and APG *in vitro* detected by pull-down assay. Amylose resin–bound MBP-APG or MBP was incubated with an equal amount of GST-PGL1. Proteins co-precipitated with the amylose resin were detected by immunoblotting using anti-GST antibody. B) *In vitro* homodimerization of APG detected by pull-down assay. Amylose resin–bound MBP-APG or MBP was incubated with an equal amount of GFP-APG extracted from *N. benthiamiana* leaves. Proteins co-precipitated with the amylose resin were detected by immunoblotting using anti-GFP antibody. C) Confocal images of interaction *in vivo* between PGL1 and APG revealed by BiFC assay in *N. benthiamiana* leaf epidermis. BF, bright field image; YFP, yellow fluorescent protein; DAPI, 4′,6-diamidino-2 phenylindole for nuclear staining; MERGE, merged view of the YFP and DAPI images. YN-PGL1+YC-APG indicates *Agrobacterium* mediated co-infiltration of constructs encoding N-EYFP-PGL1 and C-EYFP-APG (upper); YN-PGL1+YC, co-infitration of N-EYFP-PGL1 and C-EYFP alone (middle); YN+YC-APG, co-infiltration of N-EYFP alone and C-EYFP-APG (lower). (bar = 75 µm) D) Light microscopic images of homodimerization *in vivo* of APG revealed by BiFC assay. YFP was detected in the nucleus when YN-APG was co-infiltrated with YC-APG, suggesting that the protein forms a homodimer to reconstruct the YFP signal (upper). In contrast, the YFP signal was not detected when YN-APG or YC-APG was used alone. (bar = 50 µm).

PGL1 and the four candidates were translationally fused to either maltose binding protein (MBP), glutathione *S*-transferase (GST) or thioredoxin (Trx) to express soluble recombinant proteins in *E. coli* for the *in vitro* pull-down assay. We found that MBP-APG co-precipitated with GST-PGL1, suggesting the interaction of these proteins *in vitro* (Figure 2a). Co-precipitation with PGL1 was not found for the other candidates (Figure S5).

To examine the interaction between PGL1 and APG *in vivo*, we performed a bimolecular fluorescent complementation (BiFC) assay. *Agrobacterium* harboring constructs for expression of the N-terminal half of enhanced yellow fluorescent protein (EYFP) fused to PGL1 (YN-PGL1) and C-terminal half of EYFP fused to APG (YC-APG) were co-infiltrated into *Nicotiana benthamiana* leaves. The YFP fluorescence was observed in the nucleus (Figure 2c), indicating that the two proteins interact *in vivo* and are localized in the nucleus. YFP signals were not observed for the in combination of YN-PGL1 and C-EYFP (YC) or N-EYFP (YN) and YC-APG (Figure 2c), further suggesting that interaction between PGL1 and APG is necessary for reconstruction of the YFP protein.

**Figure 3:**
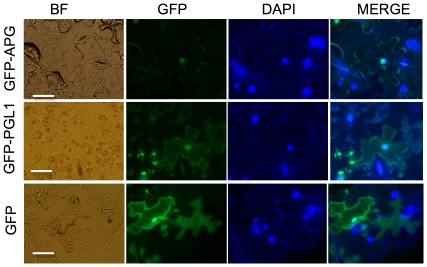
Localization of PGL1 and APG protein in plant cells. Fluorescence signal detected using a light microscope from GFP-APG (upper) and GFP-PGL1 (midle) and GFP protein (lower) expression under the 35S promoter in *N. benthiamina* leaf epidermis cells; GFP, green fluorescent; MERGE, merged view of the GFP and DAPI images. (bar = 50 µm).

We analyzed the intracellular localization of these proteins separately. *APG* and *PGL1* were fused downstream to green fluorescent protein (GFP) gene and agro-infiltrated into *N. benthamiana* leaf epidermal cells. Fluorescent signal was observed in the nucleus for GFP-APG and both the cytoplasm and nucleus for GFP-PGL1 (Figure 3), consistent with the BiFC results.

To test whether APG can form a homodimer, *Agrobacterium* harboring constructs for YN-APG and YC-APG were co-infiltrated into *N. benthamiana* leaves. YFP signals were detected in the nucleus (Figure 2d), indicating that APG is capable of forming a homodimer in plant cells. *In vitro* pull down assay also revealed that GFP-APG co-precipitated with MBP-APG (Figure 2b). Taken together, the results suggested that the APG protein is able to form both a homodimer and a heterodimer with PGL1.

A RT-PCR analysis of *APG* in the wild type showed that it is expressed in the lemma/palea and predominantly in the root (Figure S6). A previous genome-wide study of bHLH proteins categorized APG in subfamily 24 as OsbHLH106 [15].

### Suppression of *APG*, an interaction partner of *PGL1*, increases grain length

**Figure 4:**
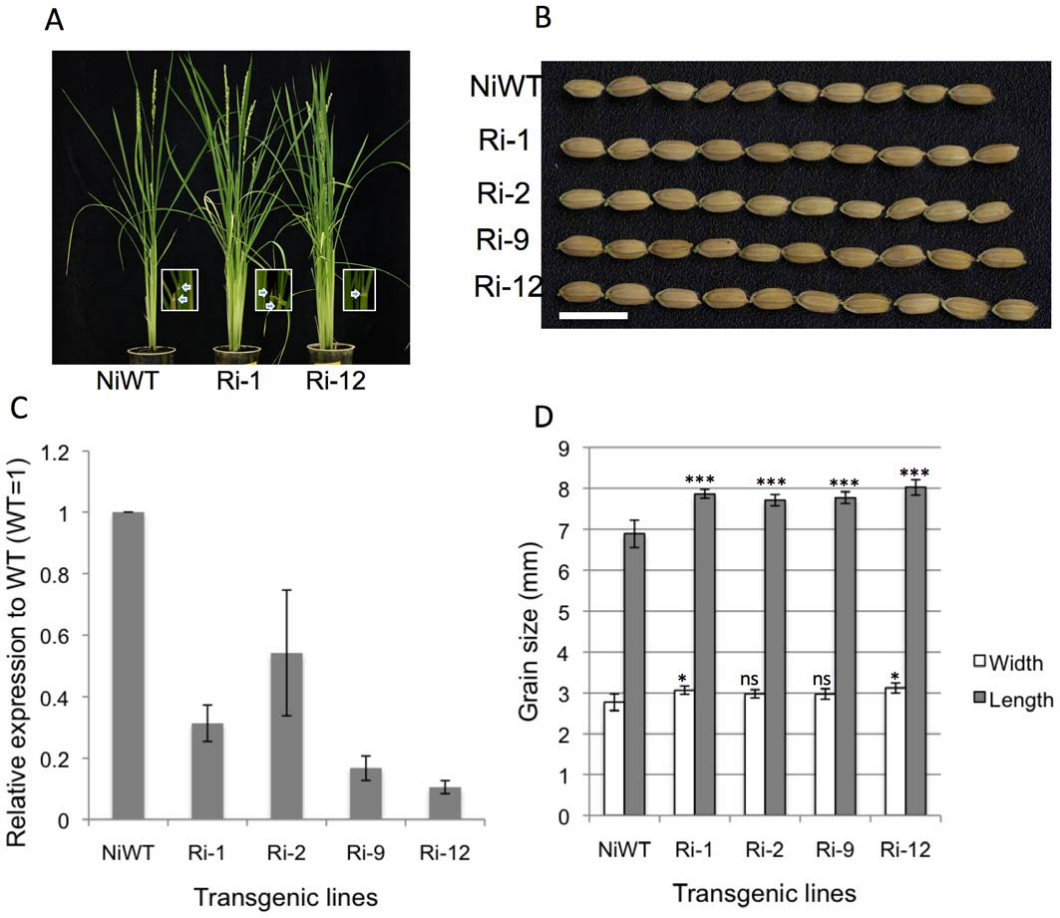
Phenotypes of APG RNAi lines. A) RNAi lines Ri-1 and Ri-12, and Nipponbare WT. Inset indicates the laminar joint leaf of each line with arrow heads (bar = 10 cm). B) Grain phenotype of representative transgenic T_0_ (bar = 1 cm). C) Quantitative PCR expression analysis of *APG* in lemma/palea of T_0_ plants (WT = 1) normalized by *OsActin*. The error bar indicates ±sd over three biological repeats. D) Comparison of grain length and width of transgenic T_0_ and wild type plant (error bar ±sd, n = 15). Asterisks denote a significant difference from the wild type as determined by Student's t tests (ns, not significant; *, p<0.05; ***,p<0.001).

Given that overexpression of *PGL1* in lemma/palea results in long grains, and that PGL1 lacks the basic domain for DNA binding while its interaction partner APG retains it, we raised the hypothesis that APG is a negative regulator of rice grain length, and its function is inhibited by PGL1 through heterodimerization. This hypothesis predicts that suppression of *APG* and overexpression of *PGL1* would give similar phenotypes. To examine this, we knocked down *APG* by the RNAi method in the Nipponbare background, and observed grain size phenotype (Figure 4a). Twenty T_0_ transgenics were produced. As expected, the *APG* RNAi lines had significantly longer grains than the wild type (Figure 4b–d). The most severely suppressed line, Ri-12, in which *APG* mRNA accumulation was ∼10% of that in the wild type, showed the longest grain length, with a 12% increase in 1000-grain weight (Table 1).

### *PGL1*/*APG-*mediated grain length is caused by elongated cells in lemma

**Figure 5:**
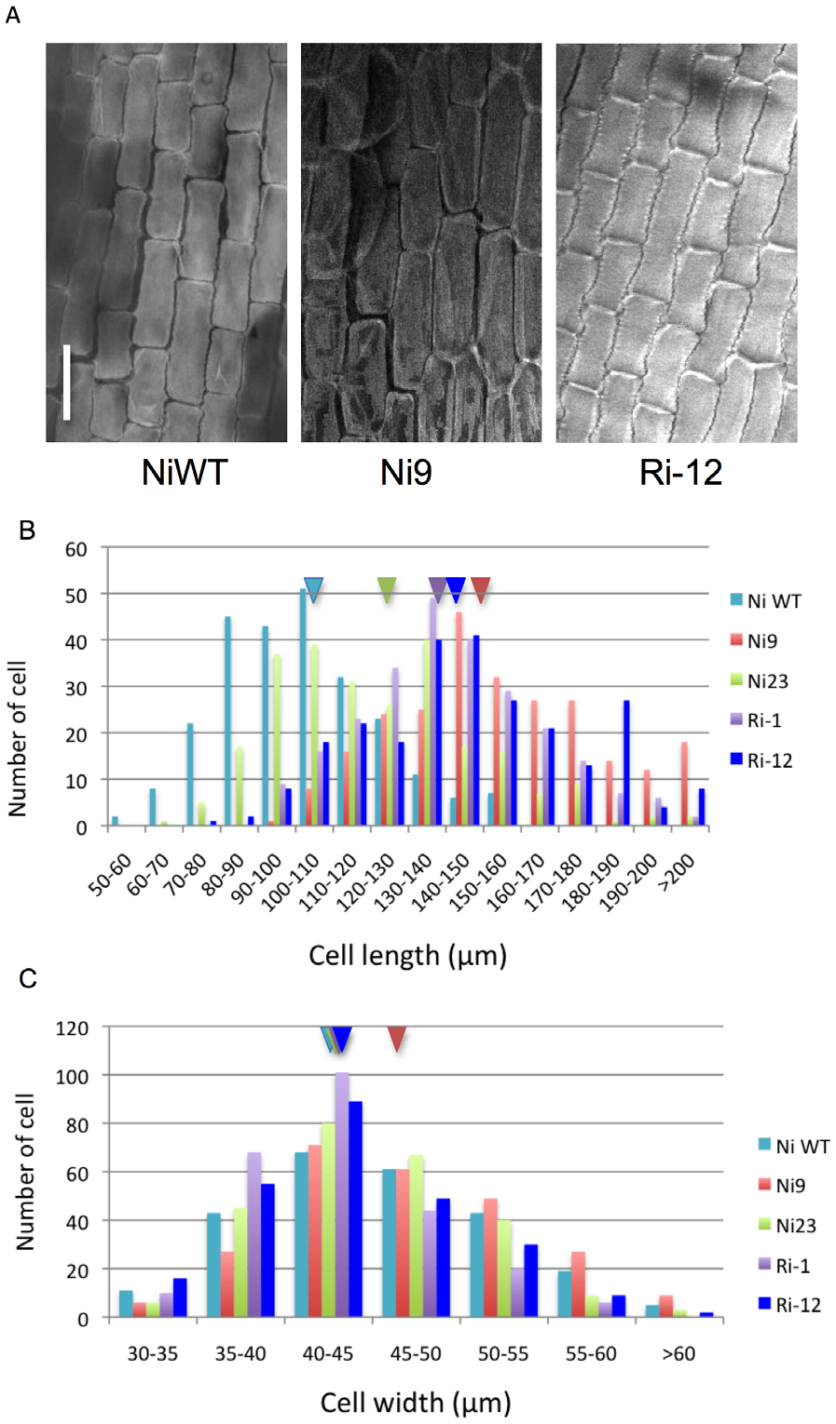
Inner epidermal cells observed by confocal microscopy. A) Lemma inner epidermal cells of NiWT and transgenic PGL1:OX (Ni9) and APG RNAi (Ri-12) (bar = 100 µm). B) Distribution of the number of cells at various cell lengths. C) Distribution of the number of cells at various cell widths; NiWT, Nippobare wild type cyan; T_0_ transgenic PGL1:OX line Ni9, red; Ni23 green; RNAi T_0_ line Ri-1, purple; and Ri-12, blue. Triangles represent average values of the respective lines.

We selected two transgenic lines with different grain sizes from APG RNAi (Ri-1 and Ri-12) and PGL1:OX (Ni9 and Ni23) to compare their lemma inner epidermal cells to those of wild type. Confocal microscopic observations revealed that the longer grain is caused by enhanced cell length (Figure 5a). Transgenic plants with long grains produced more long cells than the wild type, though the width of cells was largely unaffected (Figure 5b–c). The results were consistent between the *APG* RNAi lines and *PGL1*:OX lines of both the Nipponbare (Figure 5a–d) and Kita-ake backgrounds (Figure S2). Observation of palea inner epidermal cells of PGL1:OX (Ni9) and APG RNAi (Ri-12) showed similar results (Figure S3).

Grain filling rate is another major factor determining grain weight. We obtained three sterile PGL1:OX lines (Ni5, Ni8 and Ni10) which are unable to fill the grain. However, these lines had large lemma/palea, suggesting that the elongated grain in PGL1:OX is not related to grain filling rates (Figure S1). This is consistent with that grain size is rigidly controlled by the sizes of lemma/palea, and thus grain cannot grow to a size greater than that permitted by lemma/palea [1]–[3], [19].

### APG and PGL1 does not affect the expression of known grain length controlling genes

**Figure 6:**
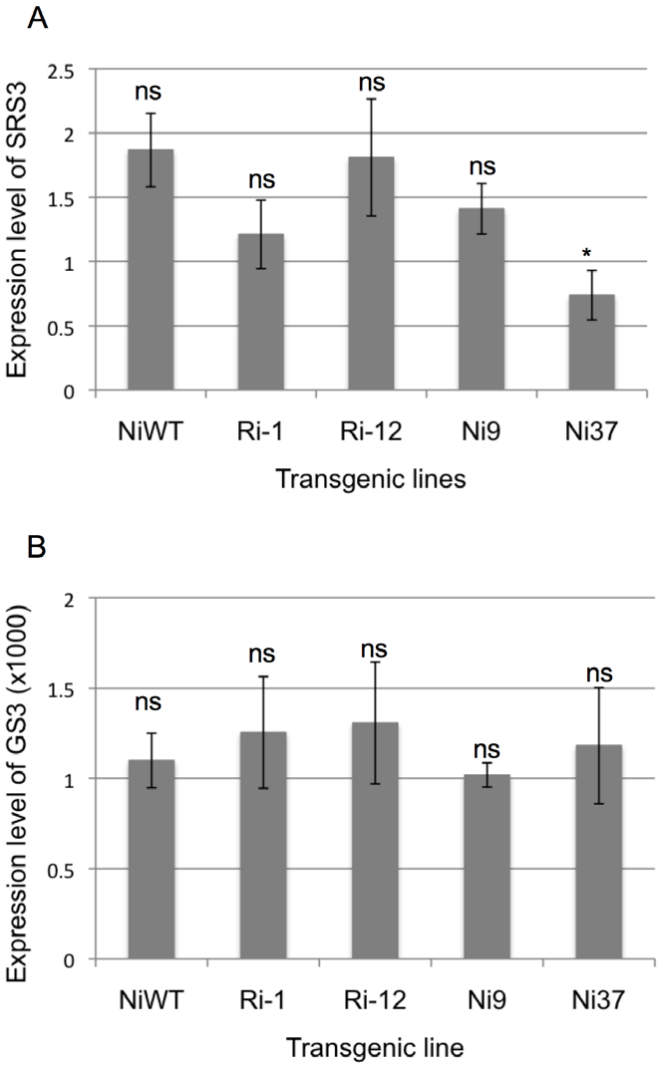
Quantitative PCR analysis of the grain length related genes *GS3* and *SRS3* in transgenic plants overexpressing PGL1 and APG RNAi T_0_ lines. A, B: Quantitave PCR analysis of the SRS3 gene (A) and GS3 (B) in lemma/palea in T_0_ transgenic plants overexpressing PGL1 and APG RNAi normalized by *OsActin*. The error bar indicates ±sd over three biological repeats. Asterisks denote a significant difference from the wild type determined by Student's t tests (ns, not significant; *, p<0.05).

To examine whether the increased grain sizes of the APG RNAi lines and PGL1:OX lines resulted from the alteration of known grain size-related genes, we analyzed expression levels of two known grain length controlling genes *SRS3* [5] and *GS3* [4] in our transgenics. qRT-PCR analysis indicated the expression of *SRS3* and *GS3* to be largely unaffected in the selected APG RNAi lines (Ri-1 and Ri-12) and PGL1:OX lines (Ni9 and Ni37) (Figure 6).

### PGL1 is a brassinosteroid-related gene

**Figure 7:**
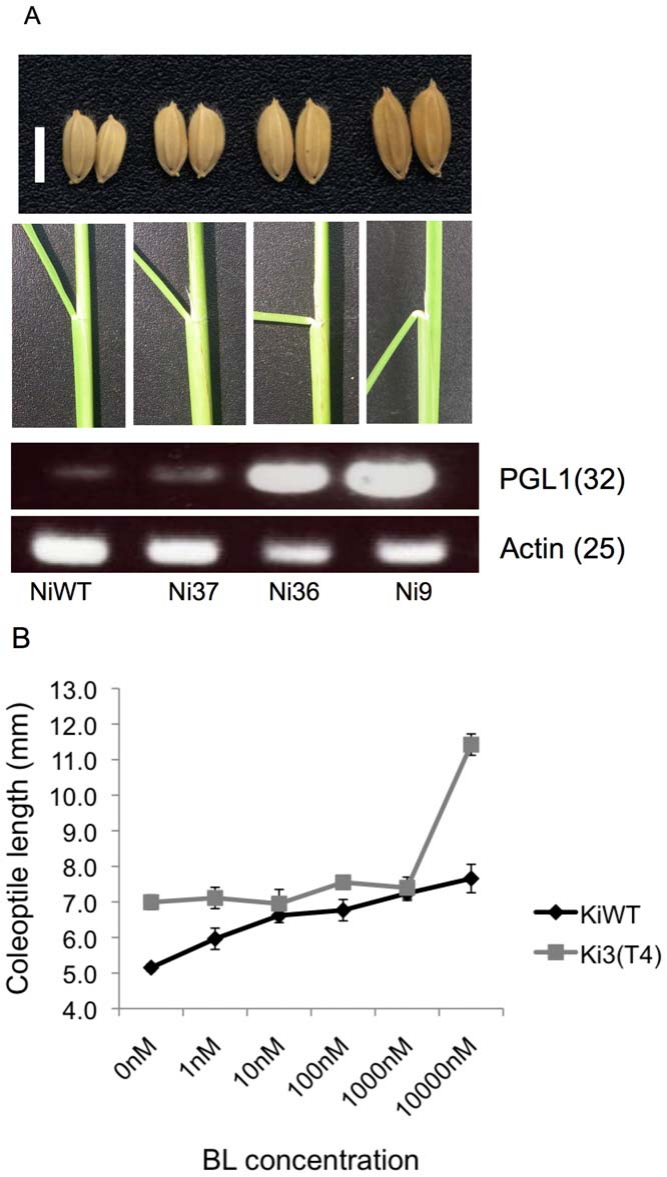
Correlation between leaf bending, grain size and expression of the PGL1 gene in lemma/palea. A) Grain phenotype (upper), Leaf bending of transgenic T_2_ and Nippobare WT plants (middle) and expression of PGL1 in lemma/palea analyzed by RT-PCR (lower). B) Coleoptile length of 5 days-old seedling growth in medium containing different concentrations of brassinolide (BL) under continuous light at 28°c of Kita-ake wild type and Ki3 T_4_ overexpressing line, (error bars indicated of ±sd, n = 15).

Overexpression of the BR signaling gene *BRASSINOSTEROID UPREGULATED1* (*BU1*), an atypical bHLH gene, increased leaf inclination [20]. In our PGL1:OX lines, we found marked leaf bending in most lines. Interestingly, levels of bending seemed to correlate to grain length and the expression level of *PGL1* in lemma/palea (Figure 7). Analysis for coleoptile length growth under different concentrations of barassinolide (BL) revealed that the PGL1:OX lines are hypersensitive to BL (Figure 7). Together, these results suggest that *PGL1* is a BR signaling gene like *BU1*. However, exogenous BL treatment had subtle or no effect on gene expression for *PGL1* and *APG*, respectively, while it enhanced *BU1* expression for ∼ three times, being consistent with previous report on *BU1* [20] (Figure S7).

Three APG RNAi lines (Ri-1, Ri-5 and Ri-13) showed obvious leaf bending (Figure 4a). This is consistent with the idea that APG and PGL1 interact and have antagonistic roles.

## Discussion

### Genetic control of grain size

Rice grain size is a major yield component, and controlled by at least four factors; grain width, length, thickness and the ability to fill the grain [21]. Findings on the genetic networks underlying these factors are very limited at present. In this study, we showed the involvement of an antagonistic pair of HLH/bHLH proteins in determining grain length; the positive regulator PGL1, an atypical bHLH, and the negative regulator APG, a typical DNA-binding bHLH.

**Figure 8:**
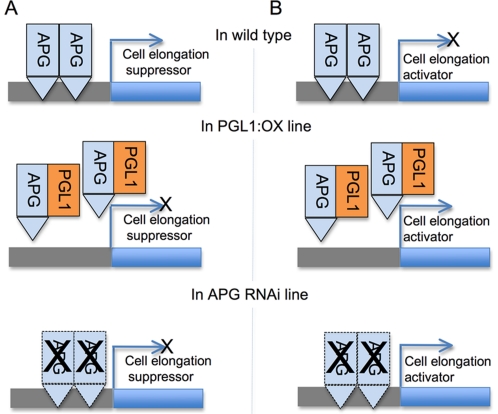
A model for grain length control by *PGL1* and *APG* in rice. A) APG is a transcriptional activator of the cell elongation suppressor gene in rice lemma/palea. APG forms homodimer or heterodimer with unidentified typical bHLH (not shown). Overexpression of PGL1 abolished the function of APG and suppressed the target gene expression allowing the cell to elongate (in the PGL1:OX line). Silencing of APG repressed the target gene expression and resulted in cell elongation (in the APG RNAi line). B) APG is a transcriptional repressor of the cell elongation activator gene in rice lemma/palea. APG forms homodimer or heterodimer with unidentified typical bHLH (not shown). In contrast to (a), the APG target gene promotes cell elongation and its function is repressed by APG in the wild type but activated in PGL1:OX and APG RNAi, allowing the cells to elongate in the lemma/palea.

To date, two grain length-controlling genes have been reported, an OSR domain-containing protein gene *GS3* and a kinesin 13 protein gene *SRS3* [4], [5]. The *srs3* mutant showed significantly decreased cell length in lemma but only slightly affected cell width (not significant at p<0.01) compared to the wild type [5]. Although alterations of neither cell size nor cell number were described for the *GS3* mechanism, the gene negatively regulates grain length and has a small positive effect on width [4]. The expression of *GS3* and *SRS3* in the PGL1:OX and APG RNAi lines was largely unaffected, suggesting that the genetic pathway through which *PGL1* and *APG* regulate grain length is independent of these genes, although the possibility of *PGL1* and *APG* being downstream of these genes can not be excluded. We also showed that the PGL1 and APG proteins interact *in vitro* and *in vivo*, and the complex of them is localized at the nucleus. bHLH proteins are reported to function either as transcription activators [22], [23] or as transcription repressors [24]. Given that the basic region of APG retains all amino acids required for binding to G-box, His 9, Glu 13 and Arg 16 [15] (Figure S6), while PGL1 lacks the basic domain required for DNA binding, APG would form a homodimer or heterodimerize with other unidentified bHLH protein, and function as a transcription factor that either activates the expression of a negative regulator of grain length or suppresses a positive regulator, while PGL1 would inhibit the effect of APG through heterodimerization (Figure 8). Taken together, our results show that the antagonistic pair of HLH/bHLH genes *PGL1* and *APG* represents a novel genetic pathway controlling rice grain size by regulating cell length in the lemma and palea.

### Homologs of *PGL1* and cell elongation in different plant species

Based on sequence homology, *PGL1* was previously classified in the *Ili* gene family consisting of seven rice genes, and named *Ili6* [13]. The *Ili* homologs of Arabidopsis were named the *PRE* gene family [13]. The members of the *Ili* and *PRE* gene families were grouped to subfamily 16 of the 32 plant bHLH subfamilies [15]. Many members of subfamily 16, such as rice ILI1 and BU1, Arabidopsis PRE1 and ATBS1, and tomato Style2.1, were reported to control cell elongation and expansion in specific organs probably through heterodimerization with other bHLH proteins. We showed that PGL1 positively controls cell length in lemma/palea. *Ili1* and *BU1* control cell elongation in rice laminar joints [13], [20]. *ATBS1* (also known as *PRE3* or *TMO7*) and *PRE1* regulate cell expansion in Arabidopsis leaves through brassinosteroid signaling [13], [14]. *Style2.1* controls cell elongation in the developing tomato style [16]. Although these atypical bHLH proteins of subfamily 16 are involved in determining cell length, their interaction partners belong to different subfamilies. PGL1 interacts with a typical bHLH protein, APG, belonging to subfamily 24, while ILI1 binds to OsIBH1 which is an atypical bHLH protein of subfamily 18 [13]. Arabidopsis PRE1 interacts with AtIBH1, a member of subfamily 18 [13]. Interaction partners of ATBS1 is an atypical bHLH protein, AIF1, belonging to subfamily 19 [14]. Functional analyses showed that each of the proteins interacting with bHLH is antagonistic [13], [14]. It is not clear why the bHLH proteins of subfamily 16 have a similar function, the elongation of cells, even though their interaction partners belong to different bHLH subfamilies. Particularly, ILI1-OsIBH1, PRE1-AtIBH1 and ATBS1-AIF are pairs of atypical bHLH proteins considered incapable of DNA binding. AIF1 is speculated to also interact with DNA binding bHLH besides ATBS1 [14]. The subfamily 16 bHLH proteins and their interactors would interact more than two bHLH proteins. Consistent with this, overexpression of rice *PGL1* (Os03g0171300) in Arabidopsis resulted in increased rosette leaf size [25], http://ricefox.psc.riken.jp/, rice FOX Arabidopsis line R90261). Clarification of the protein-protein interaction networks of subfamily 16 bHLH proteins and downstream genes regulated by these proteins would show how the members of subfamily 16 control cell elongation in different organs and species through interaction with different classes of proteins.

We found that overexpression of *PGL1* didn't affect pistil length even though a high level of transcript was accumulated in the organ (Figure S1), suggesting the function of bHLH to be dependent on different organ-specific factors. Given that *APG* is expressed in both the pistil and lemma (Figure S6), the function of APG might be strongly inhibited by an atypical bHLH other than PGL1 in the pistil. Another possibility is that the expression of downstream target gene(s) of APG is regulated differently between the lemma/palea and pistil. The identification of downstream target gene(s) of APG and other interactor(s) than PGL1 would improve our understanding of the HLH/bHLH-based regulation of cell elongation in different organs.

APG was grouped to subfamily 24 together with PIFs [15]. The bHLH domain of APG is highly homologous to that of the Arabidopsis PIF family (91% and 81% amino acid identity to PIF3 and PIF4, respectively). The bHLH domain is required for PIF4 to interact with *REPRESSOR OF GA INSENSITIVE 1–3* (*RGA1*), a DELLA protein, to regulate cell elongation in Arabidopsis [22]. However, overall amino acid identity to APG is as low as 27% for PIF3 and and 22% for PIF4. It is possible that regions of APG other than bHLH are involved in the functional difference between the PIF family and APG. For instance, The APB (active phytochrome binding) motif of PIFs is necessary for binding to phytochrome B [26], while APG has a Q to A amino acid substitution in the APB consensus sequence (Figure S6).

### Brassinosteroid-related genes and grain size

BR-related genes are reported to be important in the regulation of rice grain size [20], [27]–[29]. For instance, overexpression of the BR biosynthesis related gene *Zm-CYP-1* increased grain yield by enhancing grain filling in rice [28]. Mutations of either the BR receptor gene *OsBRI1*/*D61*, or the BR signaling gene *RGA1/D1* resulted in small grains [27], [29]–[31]. A recent study revealed that overexpression of the BR signaling gene *BU1* also increased grain size, although most of the OX lines were sterile [20]. We showed that a new probable BR signaling protein PGL1 is also involved in determining grain size through interaction with the antagonistic protein APG. Identification of target genes regulated by APG-PGL1, and other interactor of APG and PGL1 would uncover how the BR signaling pathway regulates grain size in rice.

## Materials and Methods

### Plant materials and observation of phenotypes

Rice (*Oryza sativa* L.) cv Nipponbare and Kita-ake were used for transformation as described previously [32]. Ten fertile seeds from transgenics and wild types were chosen at random for measuring grain length and width with vernier calipers. Thousand seeds weight was calculated from the weights of 200 fully fertile seeds after drying at 41°C for 1 week after harvest [28].

### Gene expression analysis by qPCR

Total RNA (2 µg) extracted from lemma/palea at the pre-anthesis stage (Methods S1) was used to synthesize first-strand cDNA with cDNA synthsis kit (Toyobo). Quantitative PCR (qPCR) for gene expression analysis was carried out with SYBR Thunderbird (Toyobo) using gene specific primers. The rice actin gene was used as a control [33]. Data were collected using an ABI PRISM 7000 sequence detection system (Applied Biosystems) and analyzed according to the instruction manual.

### Construction of plasmids

#### a) chitinase::PGL1 and APG RNAi construct

The 1685 bp upstream region of a rice chitinase gene (AB012855), hereafter refered as the chitinase promoter [17], was amplified from Nipponbare genomic DNA by PCR, fused to the 663 bp genomic sequence of *PGL1* (Os03g0171300), and inserted into a binary vector pPZP2H-Lac to create chitinase::PGL1 [34] (Methods S1, Figure 1a). The first exon of *APG* (Os05g0139100), 262 bp, was amplified from Nipponbare lemma/palea cDNA and subcloned into pHANNIBAL at *Eco*RI/*Kpn*I and *Cla*I/*Bam*HI [35] to create a hairpin structure for RNAi. The plasmid was digested with *Bam*HI and subcloned into the binary vector pBI101H-Ub to create Ubi::APG RNAi [36], [37]. The binary vectors harboring chitinase::PGL1 and Ubi::APG RNAi were introduced into *Agrobacterium tumefaciens* strains EHA101 and EHA105, respectively.

#### b) protein expression constructs

The open reading frame of each gene was amplified from Nipponbare lemma/palea cDNA by gene specific primers (Table S1). The *PGL1* fragment was subcloned into pGEX-4T.1 (GE Healthcare) to generate GST-PGL1 (Methods S1 for other candidates). The *APG* fragment was fused to the coding sequence of maltose binding protein (MBP) [38] and subcloned to pColdII (Takara).

All PCR products were sequenced before further cloning by BigDye terminator ver. 3.1 (Applied Biosystems).

### Pull down assay

The protein expression constructs were introduced into *Escherichia coli* strain BL21(DE3 pLysS) (Novagen). MBP and MBP-APG proteins were purified using amylose resin beads (New England BioLabs). GFP-APG was transiently expressed in *N. benthiamiana* leaves under the control of the 35S promoter by Agro-infiltration. The expressed GFP-APG protein was extracted by binding buffer (20 mM Tris-HCl, pH 7.5, 100 mM NaCl, 1 mM EDTA, and 1 mM DTT, 0.2% 2-mercaptoethanol). Binding was carried out as described previously [13]. MBP beads bound to MBP-APG or MBP were incubated with GST-PGL1 in binding buffer supplemented with 1× final concentration of protease inhibitor complete (Roche). The mixture was rotated at 4°C for 2 hours, and the beads were washed five times with washing buffer (20 mM Tris-HCl, pH 7.5, 300 mM NaCl, 0.05% Tween-20, 1 mM EDTA and 1 mM DTT). The proteins were eluted from the beads by heating at 65°C for 5 min in 30 µl of 2× SDS loading buffer (100 mM Tris-HCl pH 6.8, 4% SDS, 2% 2- mercaptoethanol and 20% glycerol). From each sample, 10 µl was loaded onto a 13% SDS-PAGE gel. Gel blots were reacted with anti-GST monoclonal antibody (Novagene), anti-His monoclonal antibody (Covance) or anti-GFP monoclonal antibody (MBL, Japan).

### Bimolecular fluorescence complementation (BiFC) and protein localization

*PGL1* and *APG* were amplified from Nipponbare lemma/palea cDNA and inserted into binary pBiFC vectors (Niwa, M., Daimon, Y., and Araki, T. unpublished). The same cDNA fragments were cloned into the binary vector pBINPLUS [39] for expression of the fusion proteins GFP:APG and GFP:PGL1 under the control of the 35S promoter (Methods S1). All eight possible pairwise combinations of BiFC constructs, GFP-PGL1 and GFP-APG, were transformed into *A. tumefaciens* COR308 [40]. A construct for expression of the p19 protein of tomato bushy stunt virus was used to suppress gene silencing [41]. Agrobacteria harboring BiFC constructs and GFP constructs were co-infiltrated with the p19 construct in four-weeks-old leaves of *N. benthamiana* at an OD_600_ ratio of 0.7∶0.7∶1.0 and 1.0∶1.0, respectively, for YFP/GFP localization. The plants were kept for 48 hours after infiltration under continuous light at 26°C prior to observation. Yellow fluorescence protein (YFP) and the GFP signal were visualized by confocal microscope (Leica Microsystems, Heerbrugg, Germany) or Leica DMR fluorescent microscope.

### Lemma inner epidermis cell measurement

Ten pre-anthesis florets were randomly selected. The inner epidermal layers were stained using 1 M Tris-HCl pH 9.0 with 0.1 mg/L of calcofluor (fluorescent brightener 28, Sigma-ALDRICH) and images taken under the Leica confocal microscope (Methods S1). A total of 250 random cells from 10 lemma images were measured using ImageJ software (http://rsb.info.nih.gov/ij/) for cell length and width.

## Supporting Information

Figure S1 **Homologs of** ***PGL1*** **and overexpression of** ***PGL1*** **in rice pistil.** a) Amino acid alignment of PGL1 (Os03g0171300) homologs from rice BU1 (Os06g0226500), ILI1 (Os04g0641700); Arabidopsis KDR (AT1G26945), ATBS1 or TMO7 (AT1G74500), PRE1 (AT5G39860) and tomato Style2.1 (NM001247361) using GENETYX-MAC software. The dotted line indicates the basic region, solid lines indicate helix and curve line indicates a loop region. b) RT-PCR analysis of PGL1 in Nipponbare wild type. G, genomic DNA; R, root; L,leaf; P, pistil; L/P, lemma/palea and YP, young panicle. c) Pistil phenotypes of T_0_ transgenic and wild type plants (bar = 1 mm). d) Comparison of pistil lengths of T_0_ transgenic and wild type plants, (error bar ±sd, n = 10). ns denotes no significant differences between wild type and transgenic plants as determined by Student's t tests. e) RT-PCR analysis of *PGL1* in pistils of T_0_ transgenic compared with wild type plant. f) Pistil and grain phenotypes of Nipponbare WT and the sterile line Ni10 (bar = 2 mm). g) Quantitative PCR analysis of *PGL1* in lemma/palea normalized by *OsActin*. Error bar indicates ±sd over three biological replicates.
(PPT)

Figure S2 **Overexpression of** ***PGL1*** **increased grain size in Kita-ake.** a) Quantitative PCR analysis of *PGL1* in lemma/palea of Kita-ake T_0_ plants compared with wild type plants (WT = 1) normalized by *OsActin*. Error bar indicates ±sd over three biological repeats. b) Comparison of grain length and width between Kita-ake transgenic T_0_ and T_4_ (Ki3(T_4_)) plants and the wild type (error bar indicates ±sd, n = 10). Asterisks denote a significant difference from the wild type as determined by Student's t tests (ns, not significant; ^*^, p<0.05; ^***^,p<0.001). c) Lemma inner epidermal cells of Kita-ake wild type (KiWT) and transgenic plants overexpressing *PGL1* Ki3 transgenic T_4_ (Ki3(T_4_) (bar = 100 µm). d,e) Distribution of the number of cells by cell length (d), and cell width (e); KiWT, Kita-ake wild type cyan color; transgenic T_0_ overexpressing PGL1 line Ki3, red; Ki7 green; T_4_ line Ki3(T_4_), purple. Triangles represent average values.
(PPT)

Figure S3 **Inner epidermal cells observed by confocal microscopy.** A) Palea inner epidermal cells of NiWT and transgenic PGL1:OX (Ni9) and APG RNAi (Ri-12) (bar = 150 µm). B) Distribution of the number of cells at various cell lengths. C) Distribution of the number of cells at various cell widths; NiWT, Nippobare wild type cyan; T_0_ transgenic PGL1:OX line Ni9, red; Ri-12, green. Triangles represent average values of the respective lines.
(PPT)

Figure S4 **T-DNA segregation and phenotype of Ni9 T_1_.** a) Grain phenotype of T-DNA positive (+) and negative (−) plants compared to wild type and Ni9 T_0_ plants. b) RT-PCR analysis of *PGL1* in lemma/palea of Ni9 T_1_ segregated plants compared to wild type and Ni9 T_0_ plants.
(PPT)

Figure S5 **In vitro interaction between PGL1 and other candidates revealed by pull-down assay.** a) *In vitro* interaction between GST-PGL1 and MBP-Os01g (Os01g0286100) detected by pull-down assay. Amylose resin–bound MBP-Os01g or MBP was incubated with an equal amount GST-PGL1. Proteins co-precipitated with amylose resin were detected by immunoblotting using anti-GST antibody. b) *In vitro* interaction between GST-Os04g (Os04g0618600) and MBP-PGL1. Amylose resin–bound MBP-PGL1 or MBP was incubated with an equal amount GST-04g. Proteins co-precipitated with amylose resin were detected by immunoblotting using anti-GST antibody. c) *In vitro* interaction between GST-PGL1 and Trx-Os012g (Os012g0610200). Glutathione beads bound to GST-PGL1 or GST-GFP were incubated with equal amounts Trx-Os12g. Proteins co-precipitated with glutathione beads were detected by immunoblotting using anti-His antibody.
(PPT)

Figure S6 **Genomic and amino acid structure of APG and RT-PCR analysis of APG in different tissues of Nipponbare wild type.** a) Genomic structure of the APG gene, the underline indicates the fragment used for the RNAi construct and bHLH protein domain. b) bHLH domain based alignment of APG1, PIF3 and PIF4. The dotted line indicates the basic region, solid lines indicate helix regions and curve line indicates a loop region. Asterisks (*) indicate conserved His 9, Glu 13 and Arg 16 required for binding G-box (CACGTG). c) Alignment of the N-terminal (1 to 50) amino acid sequence of APG, PIF3 and PIF4. The line indicates the APB (active phytochrome binding) motif which is required for PIF3 and PIF4 to bind to phytochrome. d) RT-PCR analysis of APG (upper), and control OsActin (lower). L, leaf; R, root; P, pistil; YP, young panicle (∼10 cm); L/P, lemma/palea; and g, genomic DNA.
(PPT)

Figure S7 **Effect of Brassinolide on PGL1 and APG expression.** Expression of *PGL1*, *APG* and *BU1* of two weeks old shoot (without root) from Nipponbare treated with 10 µm of BL or mock, Error bar indicates ±sd over three independent experiments.
(PPT)

Table S1 **List of primers used in this study.**
(DOC)

Methods S1 **Supporting Information materials and methods.**
(DOC)
