# Supplementary material for: Antagonistic Actions of HLH/bHLH Proteins Are Involved in Grain Length and Weight in Rice
Source: PLoS One. 2012 Feb 21;7(2):e31325. doi: 10.1371/journal.pone.0031325 (PMC3283642; doi:10.1371/journal.pone.0031325)
Supplement: Figure S1 — Homologs of PGL1 and overexpression of PGL1 in rice pistil. a) Amino acid alignment of PGL1 (Os03g0171300) homologs from rice BU1 (Os06g0226500), ILI1 (Os04g0641700); Arabidopsis KDR (AT1G26945), ATBS1 or TMO7 (AT1G74500), PRE1 (AT5G39860) and tomato Style2.1 (NM001247361) using GENETYX-MAC software. The dotted line indicates the basic region, solid lines indicate helix and curve line indicates a loop region. b) RT-PCR analysis of PGL1 in Nipponbare wild type. G, genomic DNA; R, root; L,leaf; P, pistil; L/P, lemma/palea and YP, young panicle. c) Pistil phenotypes of T0 transgenic and wild type plants (bar = 1 mm). d) Comparison of pistil lengths of T0 transgenic and wild type plants, (error bar ±sd, n = 10). ns denotes no significant differences between wild type and transgenic plants as determined by Student's t tests. e) RT-PCR analysis of PGL1 in pistils of T0 transgenic compared with wild type plant. f) Pistil and grain phenotypes of Nipponbare WT and the sterile line Ni10 (bar = 2 mm). g) Quantitative PCR analysis of PGL1 in lemma/palea normalized by OsActin. Error bar indicates ±sd over three biological replicates. (PPT) [file pone.0031325.s001.ppt]

## Slide 1
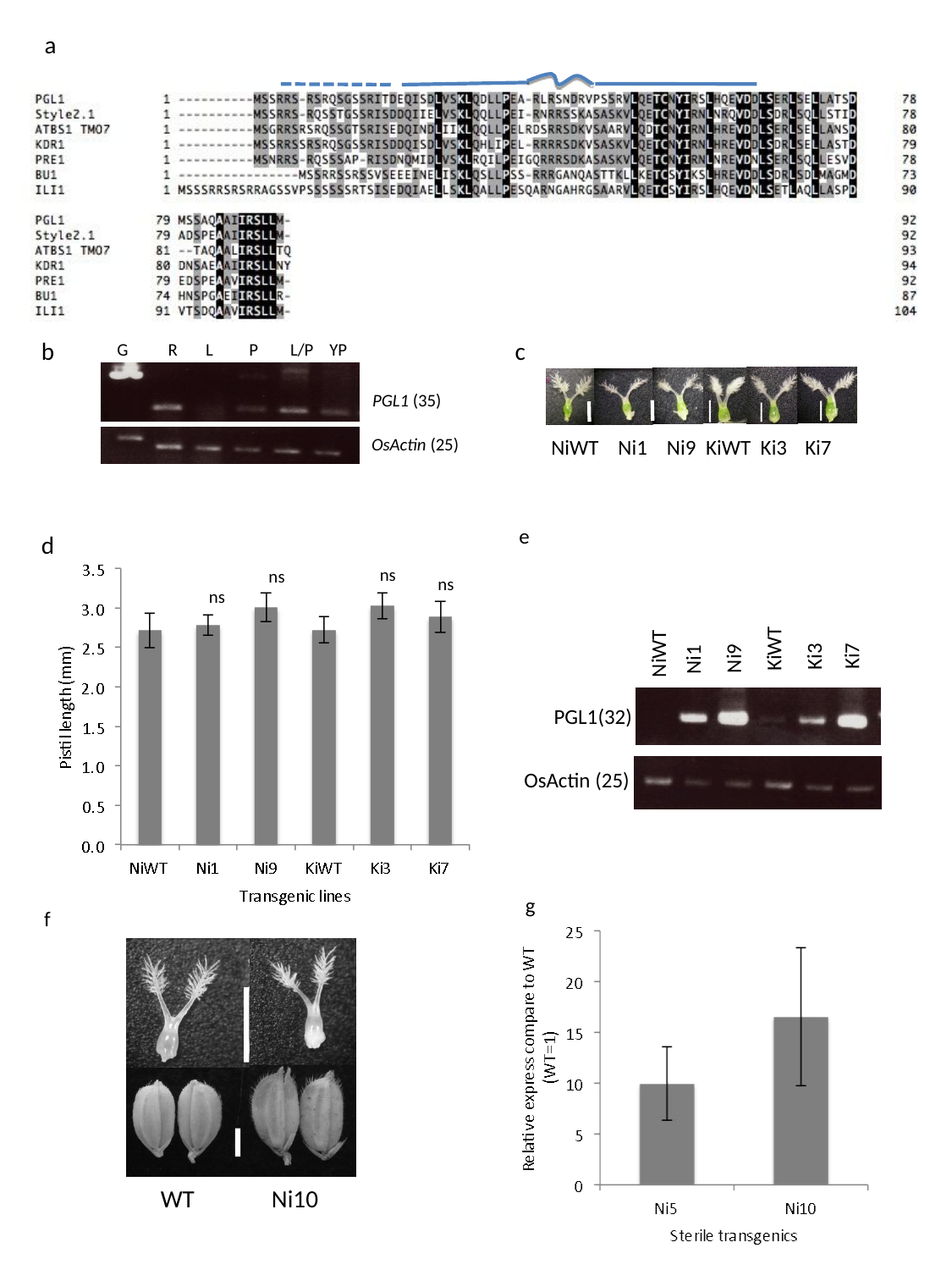

a
b
c
G R L P L/P YP
PGL1 (35)
OsActin (25)
NiWT Ni1 Ni9 KiWT Ki3 	Ki7
e
d
ns
ns
ns
ns
KiWT
NiWT
Ki7
Ki3
Ni9
Ni1
PGL1(32)
OsActin (25)
g
f
WT Ni10
