# Supplementary figures and images for: Antagonistic Actions of HLH/bHLH Proteins Are Involved in Grain Length and Weight in Rice
Source: PLoS One. 2012 Feb 21;7(2):e31325. doi: 10.1371/journal.pone.0031325 (PMC3283642; doi:10.1371/journal.pone.0031325)

## Slide 1
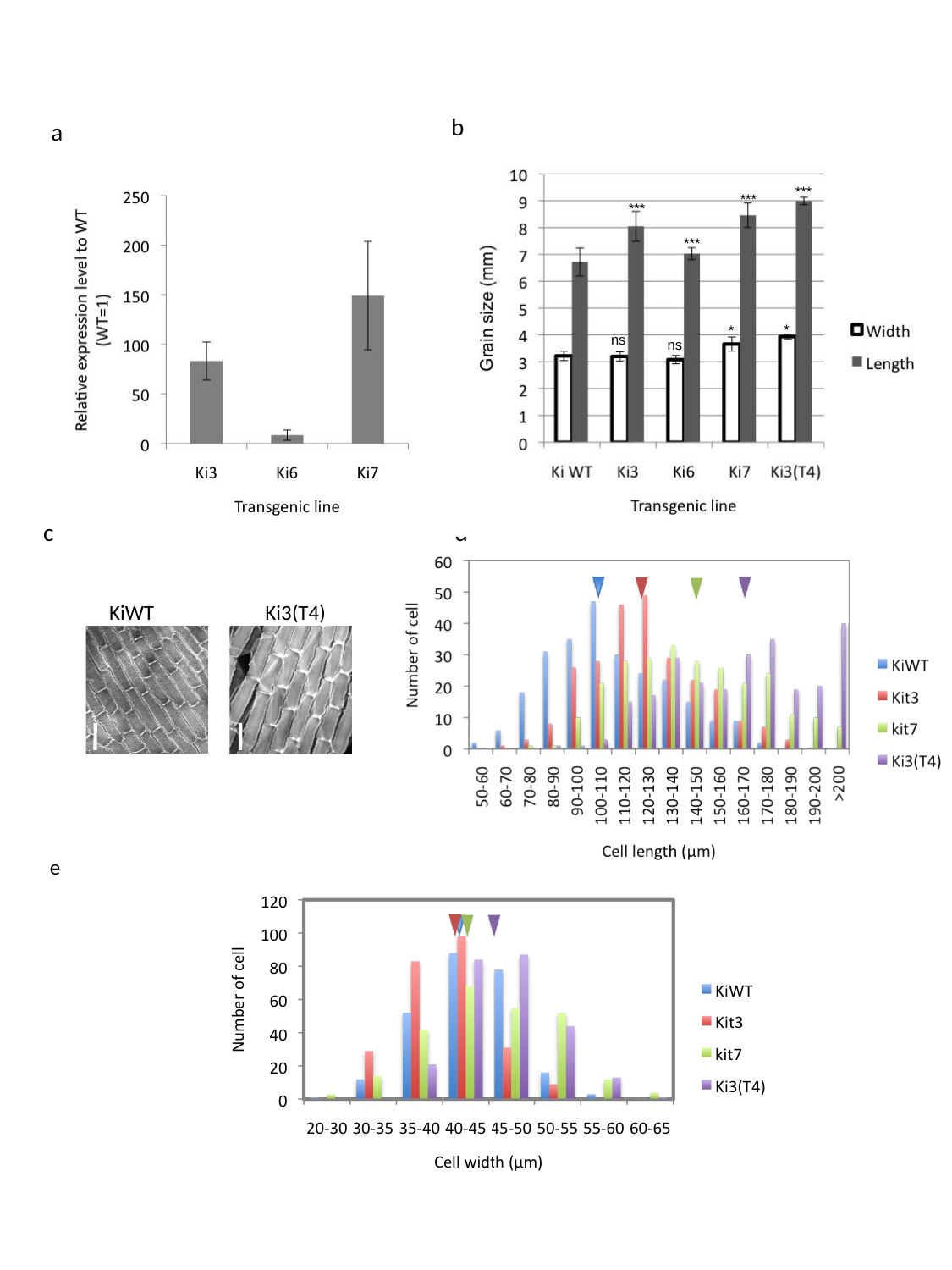

b
a
***
***
***
***
*
*
ns
ns
c
d
KiWT		 Ki3(T4)
e

Supplement: Figure S2 — Overexpression of PGL1 increased grain size in Kita-ake. a) Quantitative PCR analysis of PGL1 in lemma/palea of Kita-ake T0 plants compared with wild type plants (WT = 1) normalized by OsActin. Error bar indicates ±sd over three biological repeats. b) Comparison of grain length and width between Kita-ake transgenic T0 and T4 (Ki3(T4)) plants and the wild type (error bar indicates ±sd, n = 10). Asterisks denote a significant difference from the wild type as determined by Student's t tests (ns, not significant; *, p<0.05; ***,p<0.001). c) Lemma inner epidermal cells of Kita-ake wild type (KiWT) and transgenic plants overexpressing PGL1 Ki3 transgenic T4 (Ki3(T4) (bar = 100 µm). d,e) Distribution of the number of cells by cell length (d), and cell width (e); KiWT, Kita-ake wild type cyan color; transgenic T0 overexpressing PGL1 line Ki3, red; Ki7 green; T4 line Ki3(T4), purple. Triangles represent average values. (PPT) [file pone.0031325.s002.ppt]

## Slide 1
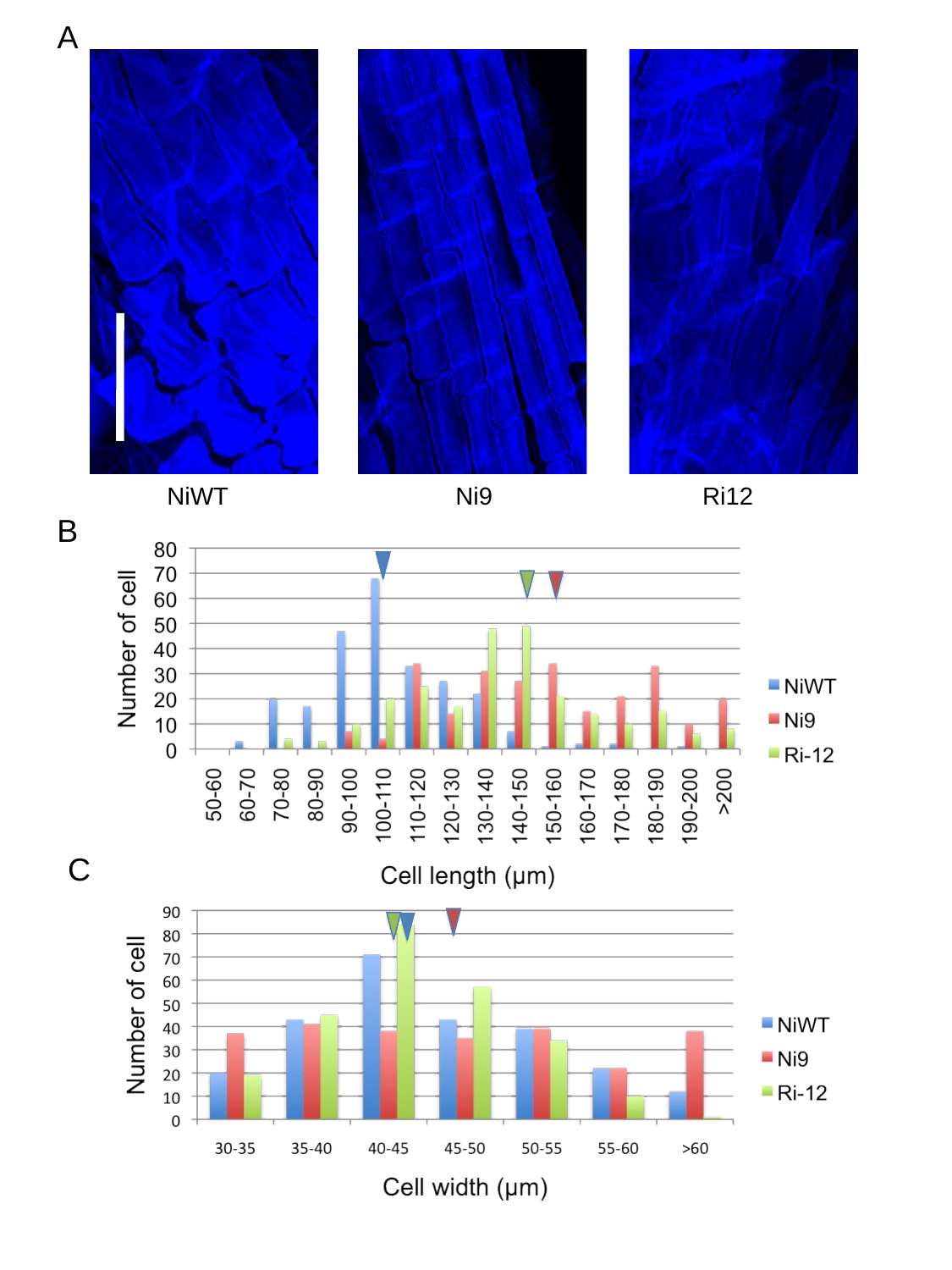

A
NiWT				 Ni9			 Ri12
B
C

Supplement: Figure S3 — Inner epidermal cells observed by confocal microscopy. A) Palea inner epidermal cells of NiWT and transgenic PGL1:OX (Ni9) and APG RNAi (Ri-12) (bar = 150 µm). B) Distribution of the number of cells at various cell lengths. C) Distribution of the number of cells at various cell widths; NiWT, Nippobare wild type cyan; T0 transgenic PGL1:OX line Ni9, red; Ri-12, green. Triangles represent average values of the respective lines. (PPT) [file pone.0031325.s003.ppt]

## Slide 1
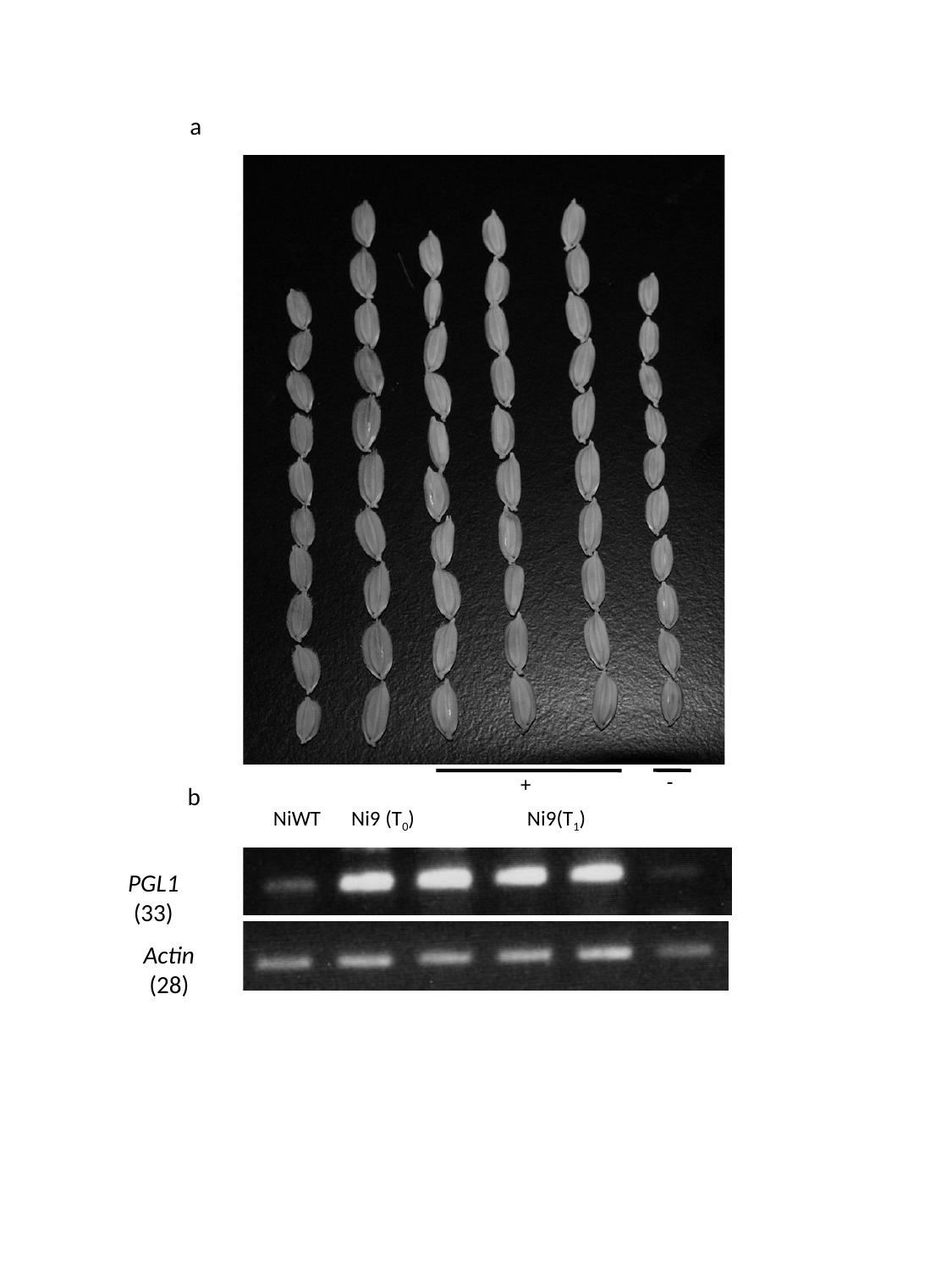

a
NiWT	 Ni9 (T0)		Ni9(T1)
PGL1
(33)
Actin
(28)
-
+
b

Supplement: Figure S4 — T-DNA segregation and phenotype of Ni9 T1. a) Grain phenotype of T-DNA positive (+) and negative (−) plants compared to wild type and Ni9 T0 plants. b) RT-PCR analysis of PGL1 in lemma/palea of Ni9 T1 segregated plants compared to wild type and Ni9 T0 plants. (PPT) [file pone.0031325.s004.ppt]

## Slide 1
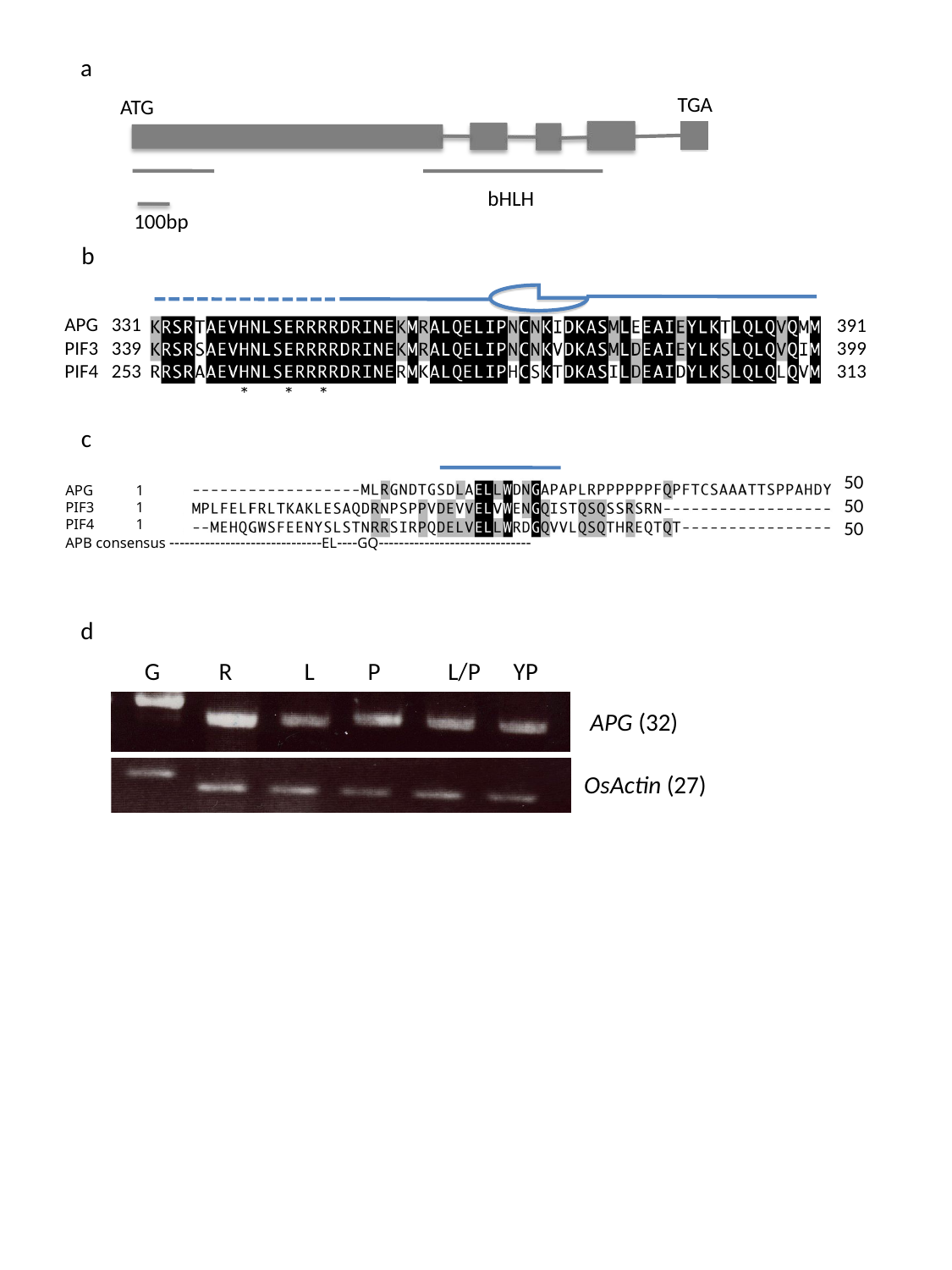

a
TGA
ATG
bHLH
100bp
b
APG 331
PIF3 339
PIF4 253
 391
 399
 313
* * *
c
 50
 50
 50
APG 	 1
PIF3 	 1
PIF4 	 1
APB consensus ------------------------------EL----GQ------------------------------
d
G	 R	 L	 P	 L/P YP
OsActin (27)
APG (32)

Supplement: Figure S6 — Genomic and amino acid structure of APG and RT-PCR analysis of APG in different tissues of Nipponbare wild type. a) Genomic structure of the APG gene, the underline indicates the fragment used for the RNAi construct and bHLH protein domain. b) bHLH domain based alignment of APG1, PIF3 and PIF4. The dotted line indicates the basic region, solid lines indicate helix regions and curve line indicates a loop region. Asterisks (*) indicate conserved His 9, Glu 13 and Arg 16 required for binding G-box (CACGTG). c) Alignment of the N-terminal (1 to 50) amino acid sequence of APG, PIF3 and PIF4. The line indicates the APB (active phytochrome binding) motif which is required for PIF3 and PIF4 to bind to phytochrome. d) RT-PCR analysis of APG (upper), and control OsActin (lower). L, leaf; R, root; P, pistil; YP, young panicle (∼10 cm); L/P, lemma/palea; and g, genomic DNA. (PPT) [file pone.0031325.s006.ppt]

## Slide 1
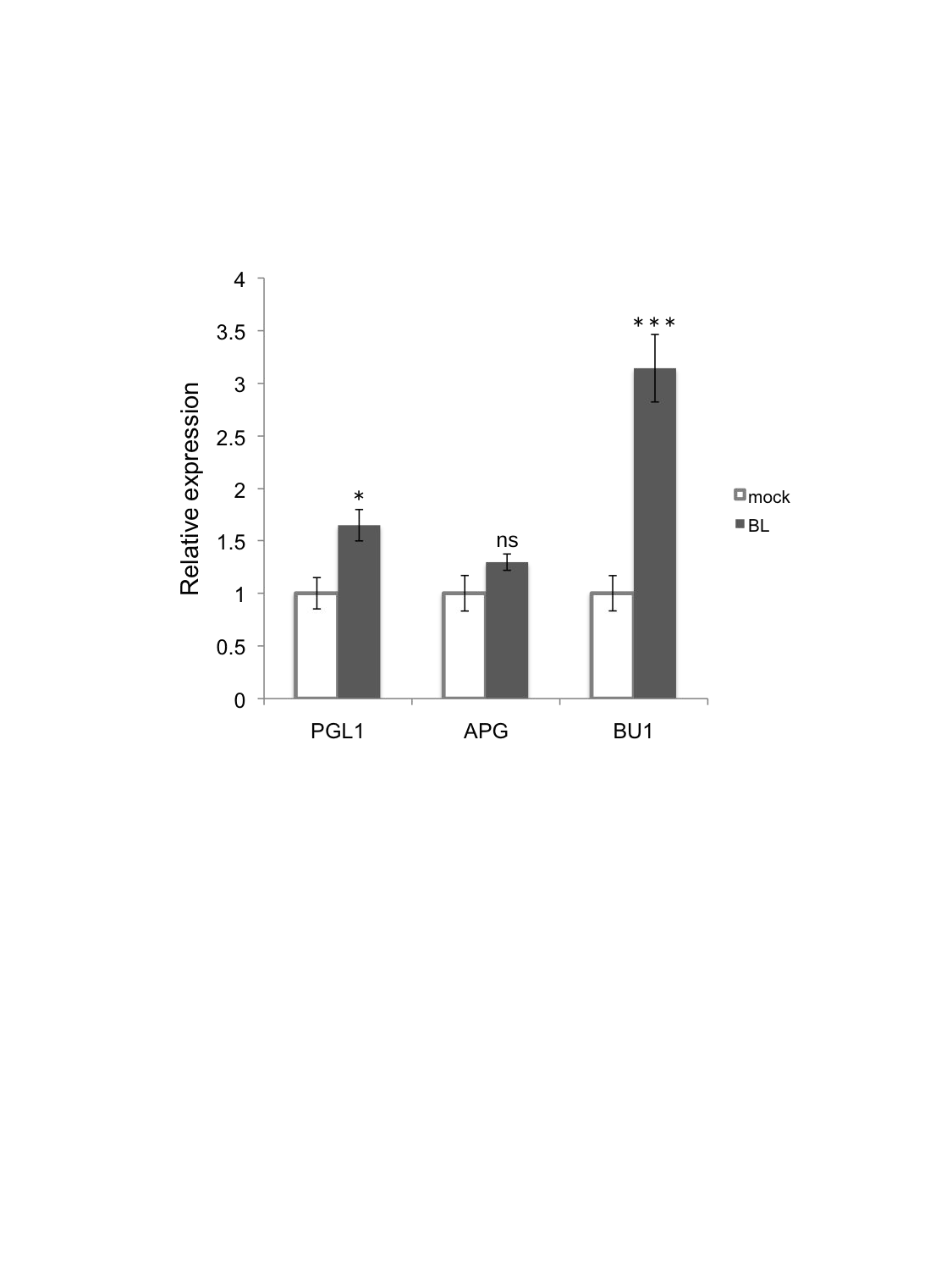

***
*
ns

Supplement: Figure S7 — Effect of Brassinolide on PGL1 and APG expression. Expression of PGL1, APG and BU1 of two weeks old shoot (without root) from Nipponbare treated with 10 µm of BL or mock, Error bar indicates ±sd over three independent experiments. (PPT) [file pone.0031325.s007.ppt]
