# Supplementary material for: Antagonistic Actions of HLH/bHLH Proteins Are Involved in Grain Length and Weight in Rice
Source: PLoS One. 2012 Feb 21;7(2):e31325. doi: 10.1371/journal.pone.0031325 (PMC3283642; doi:10.1371/journal.pone.0031325)
Supplement: Figure S5 — In vitro interaction between PGL1 and other candidates revealed by pull-down assay. a) In vitro interaction between GST-PGL1 and MBP-Os01g (Os01g0286100) detected by pull-down assay. Amylose resin–bound MBP-Os01g or MBP was incubated with an equal amount GST-PGL1. Proteins co-precipitated with amylose resin were detected by immunoblotting using anti-GST antibody. b) In vitro interaction between GST-Os04g (Os04g0618600) and MBP-PGL1. Amylose resin–bound MBP-PGL1 or MBP was incubated with an equal amount GST-04g. Proteins co-precipitated with amylose resin were detected by immunoblotting using anti-GST antibody. c) In vitro interaction between GST-PGL1 and Trx-Os012g (Os012g0610200). Glutathione beads bound to GST-PGL1 or GST-GFP were incubated with equal amounts Trx-Os12g. Proteins co-precipitated with glutathione beads were detected by immunoblotting using anti-His antibody. (PPT) [file pone.0031325.s005.ppt]

## Slide 1
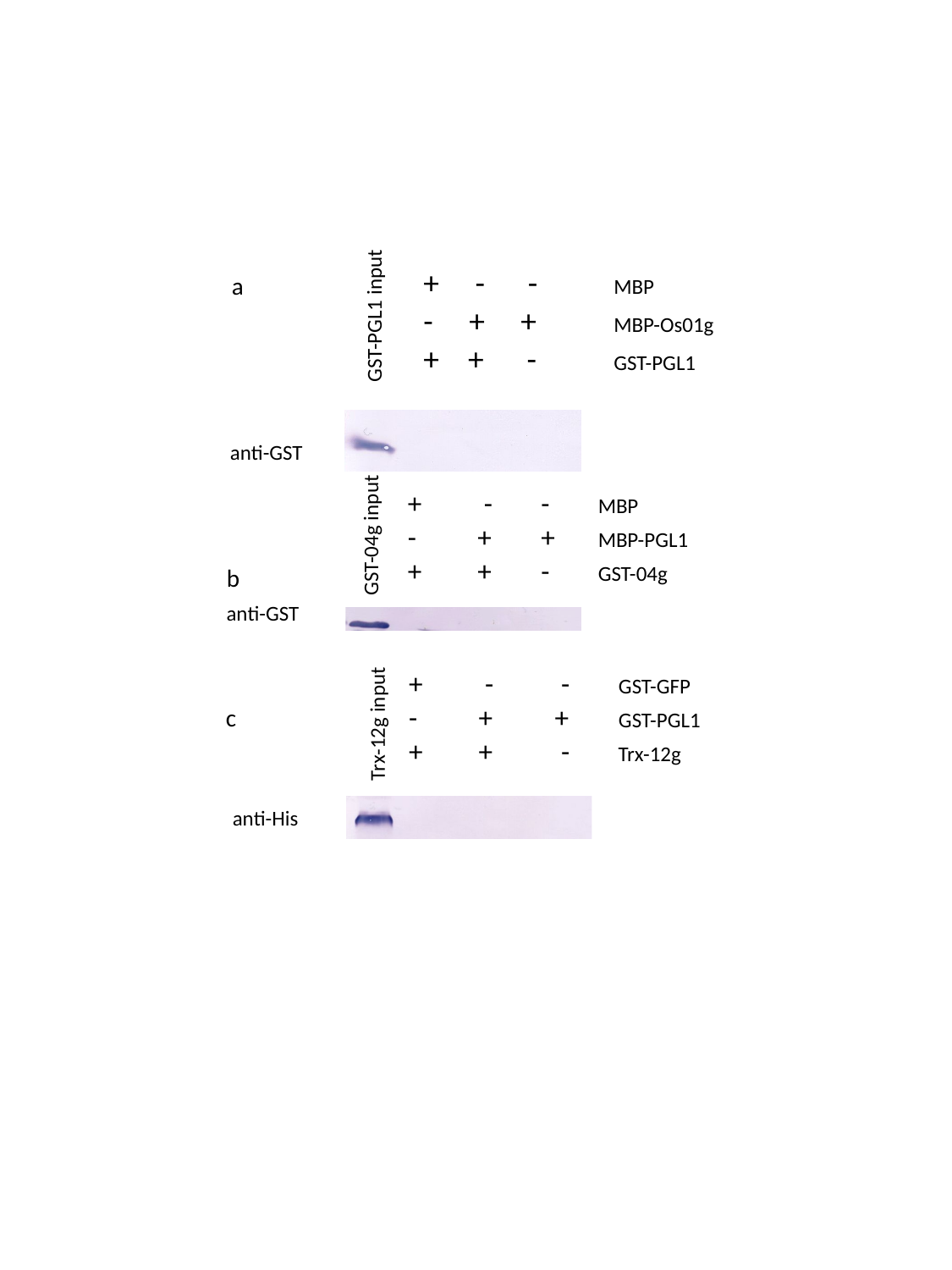

GST-PGL1 input
+ - -		MBP
- + +		MBP-Os01g
+ + -		GST-PGL1
anti-GST
a
GST-04g input
+ 	 - 	 -	MBP
- 	 + 	 +	MBP-PGL1
+ 	 + 	 -	GST-04g
anti-GST
b
Trx-12g input
+ 	 - 	 -	 GST-GFP
- 	 + 	 +	 GST-PGL1
+ 	 + 	 -	 Trx-12g
anti-His
c
