# Supplementary material for: Antagonistic Actions of HLH/bHLH Proteins Are Involved in Grain Length and Weight in Rice
Source: PLoS One. 2012 Feb 21;7(2):e31325. doi: 10.1371/journal.pone.0031325 (PMC3283642; doi:10.1371/journal.pone.0031325)
Supplement: Table S1 — List of primers used in this study. (DOC) [file pone.0031325.s008.doc]

| **Name** | **Sequence** | **Purpose** |
| --- | --- | --- |
| FLO2IM | ATGTCAAGCCGGAGGTCACG | qPCR analysis |
| RLO2IV | CGACTCCGGAGCAATGATAGA | qPCR analysis |
| FChitiH | CCCAAGCTTGTTATGCTCGTTTTGCTTAT | chitinase prometer |
| RChitiK | GGGGTACCGGCAAGATGCTTATTTCT | chitinase prometer |
| FPGL1K | GGGGTACCATGTCAAGCCGGAGGTCAC | amplify coding and RT-PCR |
| RPGL1B | CGGGATCCCTACATCAGAAGGCTGCGGA | amplify coding and RT-PCR |
| OsACT1U | TCCATCTTGGCATCTCTCAG | Actin for RT-PCR |
| OsACT1L | GTACCCGCATCAGGCATCTG | Actin for RT-PCR |
| Actin1_F | CCCTCCTGAAAGGAAGTACAGTGT | Actin for qPCR |
| Actin1_R | GTCCGAAGAATTAGAAGCATTTCC | Actin for qPCR |
| FOs12gEco | GGAATTCATGAACCAGTTCGTCCCTGAT | Protein expression construct |
| ROs12gXho | CCCTCGAGTCAGGAGTCAGCGGCTGCG | Protein expression construct |
| FOs01gEco | GGAATTCATGTCCGACGGCAACGACTT | Protein expression construct |
| ROs01gXho | CCCTCGAGTTATGTTTCAGCCCCATCTCT | Protein expression construct |
| FAPGEco | GGAATTCATGCTACGCGGGAACGACAC | Protein expression construct |
| RAPGSma | CCCCGGGTCACGCCTGCTTCACGGC | Protein expression construct |
| FOs04gBam | CGGGATCCATGGAAGCAAGAAGGCCTAC | Protein expression construct |
| ROs04gXho | CCCTCGAGTCACTCCAAGAAGTTGTACG | Protein expression construct |
| FOs07gEco | GGAATTCATGGATGCGGGTGCAACTGC | Protein expression construct |
| ROs07gXho | CCCTCGAGTCAAGCTGAGTCCACACAGC | Protein expression construct |
| FAPGRNai | GGGGTACCATCGATGCTACGCGGGAACGAC | Os05g RNAi construct |
| RAPGRNai | GGAATTCGGATCCACGACAGGGTGGTAATG | Os05g RNAi construct |
| FAPGa | GCGTCATGAACTTCACCTTCTTCTC | Os05g qPCR analysis |
| RAPGb | ATCGACATCATCTGCACCTGCA | Os05g qPCR analysis |
| GS3aF | GAACTCCTGATCCATTCATAACGA | GS3 qPCR analysis |
| GS3bR | GAAACTTCTTCAAGAAGTGGTGAG | GS3 qPCR analysis |
| FSRS3 | CTCTTCTATGGAACCTGACAG | SRS3 qPCR analysis |
| RSSR3qP2 | GGTTTACTGTTGTAGCTAATTCG | SRS3 qPCR analysis |
| Fhpt1 | ATGAAAAAGCCTGAACTCACCGCGA | HPT gene for probe |
| Rhpt1 | CTATTTCTTTGCCCTCGGACGAGT | HPT gene for probe |
| FNdMalE | TAATTAACATATGAAAATCGAAGAAGGTAAAC | MBP gene for MBP pCold construct |
| RBmMalE | AGAGGATCCGAATTCTGAAATC | MBP gene for MBP pCold construct |
| FAPGXb | GCTCTAGAATGCTACGCGGGAACGACAC | GFP-construct, BiFC |
| RAPGBa | CGGGATCCTCACGCCTGCTTCACGGC | GFP construct, BiFC |
| RAPGStopB | CGGGATCCCGCCTGCTTCACGGCGGG | BiFC |
| FPGL1Xb | GCTCTAGAATGTCAAGCCGGAGGTCAC | GFP construct, BiFC |
| RPGL1StopB | CGGGATCCCATCAGAAGGCTGCGGATGAT | BiFC |
