# Supplementary material for: Antagonistic Actions of HLH/bHLH Proteins Are Involved in Grain Length and Weight in Rice
Source: PLoS One. 2012 Feb 21;7(2):e31325. doi: 10.1371/journal.pone.0031325 (PMC3283642; doi:10.1371/journal.pone.0031325)
Supplement: Methods S1 — Supporting Information materials and methods. (DOC) [file pone.0031325.s009.doc]

**Supporting Information materials and methods**

**Genomic DNA and gene expression analysis**

Genomic DNA was extracted from young leaves with the cetyltrimethylammonium bromide method [1]. Transgenic plants were first tested by PCR followed by Southern blotting. Genomic DNA of each transgenic plant was digested with *Hin*dIII. The hygromycin phosphotransferase (*HPT*) gene was amplified from pPZP2H-Lac [2] and used as a probe with DIG-PCR labeling (Roche) for Southern blotting.

Lemma/palea and pistils at the pre-anthesis stage, leaves and roots of one-week old plants were separated and subjected to RNA extraction with a RNeasy plant mini kit (Qiagen) and treated with DNase (Wako) followed by phenol chloroform purification and stored at -80°C until used. Gene specific primers (Table S1) and rice the actin gene were used as a control for RT-PCR [3]. The amplification was performed as follows: 2min at 94°C, 32 cycles of 15s at 94°C, 15s at 60°C and 15s at 72°C, followed by 5min at 72°C as the final extension.

**Plasmid construction**

*a) chitinase::PGL1 construct*

*PGL1* and chitinase promoter fragments were sub-cloned into the pHK7 vector [4] at *Kpn*I/*Bam*HI and *Hin*dIII*/Kpn*I, respectively. The expression cassette was digested with *Hin*dIII and *Sma*I to obtain the chitinase promoter, and *PGL1* with the nopaline synthase (NOS) terminator was moved to the binary vector pPZP2H-Lac [2] (Fig1a). The binary vector was introduced into *Agrobacterium tumefaciens* strain EHA101 by electroporation.

*b) protein expression construct*

*PGL1* and *Os04g* (Os04g0618600) fragments were sub-cloned into pGEX-4T.1 (GE Healthcare) at *Eco*RI/*Xho*I and *Eco*RI/*Bam*HI to make GST-PGL1 and GST-Os04g, respectively. To generate MBP-PGL1, the PGL1 fragment was sub-cloned into pDW363 at *Sac*I/*Bam*HI [5].

We found that some of the candidate proteins were degraded during induction. Low temperature induction is a promising way to keep a protein intact and thus we modified the vector constructs. First, the *MBP* gene (without stop codon) was amplified by PCR from pDW363 with gene specific primers (Table S1) and sub-cloned into pColdII (Takara) at *Nde*I and *Bam*HI site. Then, *APG* and *Os01g* (Os01g0286100) fragments were sub-cloned to multiple cloning sites of pColdII vector at *EcoR*I/*Sma*I and *Eco*RI/*Xho*I to generate MBP-APG and MBP-Os01g, respectively. The *Os12g* (Os012g0610200) fragment was sub-cloned into pET32a (Novagen) at *Eco*RI/*Xho*I to generate Trx-Os12g.

*c) BiFC construct:*

PGL1 and APG (with stop codon) were fused to the N-terminal half of EYFP or C-terminal half of EYFP at *Xba*I*/Xho*I and *Xba*I*/Kpn*I, respectively in pBI N-NY and pBI N-CY (Niwa, M., Daimon, Y., and Araki, T. unpublished). The plasmids generated from PGL1 were YN-PGL1 and YC-PGL1. For APG, YN-APG and YC-APG were produced.

PGL1 and APG (without stop codon) were sub-clone into pBI C-NY or pBI C-CY at *Xba*I and *Bam*HI sites. N-terminal half of EYFP or C-terminal half of EYFP was fused to the C-terminal of the target protein in these plasmids. The plasmids generated from PGL1 were PGL1-YN and PGL1-YC. Plasmids generated from APG were APG-YN and APG-YC.

*d) GFP fusion construct*

Coding regions of *PGL1* and *APG* were fused to downstream of GFP gene [6] and cloned into a binary vector pBINPLUS [7]. CaMV 35S promoter with *ADH* enhancer sequence [8] and nopaline synthase terminator were inserted to the binary vectors for expression of fusion proteins GFP:APG and GFP:PGL1.

**Brassinolide induction in coleoptiles elongation**

Kita-ake wild type and Ki3 (T_4_) seeds were surface sterilized with 2 % of NaClO (Wako) and grown on medium (hormone free N6 medium, [9]) containing different concentration of brassinolide (BL). The plants were grown under continuous light at 28 °C for 5 days. Images of coleoptiles from each treatment were taken and length was measured. BL treatment was carried out as described in [10]. Dehusked Nipponbare seeds were surface sterilized with 2 % of NaClO (Wako) and grown on medium (hormone free N6 medium, [9]). Ten days-old seedlings were transferred to liquid medium. After 3 days, seedlings were incubated with 10 μM of BL (Daiichi Fine Chemical) or mock (without BL) for 24 hours before harvesting mRNA for qPCR analysis.

**Inner epidermal cell observation**

Ten florets at pre-anthesis were fixed with FAA overnight then washed with 100 % ethanol several times and kept in 70 % ethanol for long-term storage until use. The middle of each lemma was excised to obtain a uniform place for observation. 1 N NaOH was added to the excised sample and boiled in hot water for 5 min. The samples were transferred to staining solution containing 1 M Tris-HCl pH 9.0 with 0.1 mg/L of calcofluor (Fluorescent Blightner 28, Sigma-ALDRICH) and kept in dark for 12 hours before confocal microscope observation (Leica Microsystems, Heerbrugg, Germany).

**References for support information**

1. Murray MG, Thompson WF. (1980) Rapid isolation of high molecular weight plant DNA. Nucleic Acids Res 8: 4321-4325.

2. Fuse T, Sasaki T, Yano M. (2001) Ti-plasmid vectors useful for functional analysis of rice genes. Plant Biotechnol 18: 219-222.

3. Kojima S, Takahashi Y, Kobayashi Y, Monna L, Sasaki T, et al. (2002) *Hd3a*, a rice ortholog of the Arabidopsis *FT* gene, promotes transition to flowering downstream of *Hd1* under short-day conditions. Plant Cell Physiol 43: 1096-1105.

4. Harikrishna K, Jampates-Beale R, Milligan SB, Gasser CS. (1996) An endochitinase gene expressed at high levels in the stylar transmitting tissue of tomatoes. Plant Mol Biol 30: 899-911.

5. Tsao KL, DeBarbieri B, Michel H, Waugh DS. (1996) A versatile plasmid expression vector for the production of biotinylated proteins by site-specific, enzymatic modification in *Escherichia coli.* Gene 169: 59-64.

6. Chiu W, Niwa Y, Zeng W, Hirano T, Kobayashi H, et al. (1996) Engineered GFP as a vital reporter in plants. Curr Biol 6: 325-330.

7. van Engelen FA, Molthoff JW, Conner AJ, Nap JP, Pereira A, et al. (1995) pBINPLUS: An improved plant transformation vector based on pBIN19. Transgenic Res 4: 288-290.

8. Sugio T, Satoh J, Matsuura H, Shinmyo A, Kato K. (2008) The 5'-untranslated region of the *Oryza sativa* alcohol dehydrogenase gene functions as a translational enhancer in monocotyledonous plant cells. J Biosci Bioeng 105: 300-302.

9. Hiei Y, Komari T. (2008) Agrobacterium-mediated transformation of rice using immature embryos or calli induced from mature seed. Nat Protoc 3: 824-834.

10. Tanaka A, Nakagawa H, Tomita C, Shimatani Z, Ohtake M, et al. (2009) *BRASSINOSTEROID UPREGULATED1*, encoding a helix-loop-helix protein, is a novel gene involved in brassinosteroid signaling and controls bending of the lamina joint in rice. Plant Physiol 151: 669-680.
